# Artificial Intelligence‐Powered One Health: A Predictive Framework for Managing Persistent Chemical Threats across Human, Animal, and Environmental Systems

**DOI:** 10.1002/gch2.202500607

**Published:** 2026-08-30

**Authors:** Jose L. Domingo

**Affiliations:** ^1^ School of Medicine, Laboratory of Toxicology and Environmental Health Universitat Rovira i Virgili Reus Catalonia Spain

**Keywords:** artificial intelligence, exposome, one health, PFAS, POPs, predictive toxicology

## Abstract

The increasing burden of persistent organic pollutants, per‐ and polyfluoroalkyl substances, heavy metals, and emerging contaminants represents a global threat to human, animal, and ecosystem health. Current management strategies remain fragmented and reactive, limiting the effectiveness of the One Health paradigm. This review introduces an artificial intelligence (AI)‐powered One Health framework designed to transition from surveillance to predictive prevention. Using a literature review across PubMed, Scopus, Google Scholar, and Web of Science (up to October 2025), recent advances in machine learning, deep learning, geospatial AI, and physics‐informed models for chemical risk assessment are synthesized. The proposed framework integrates four core capabilities: (1) geospatial AI for real‐time source tracking and fate modeling, (2) multispecies exposome reconstruction for unified exposure assessment, (3) predictive toxicology engines enabling cross‐species risk extrapolation, and (4) AI‐driven optimization of remediation and policy scenarios. Ethical and governance challenges, including algorithmic bias, transparency, and environmental justice, are critically examined. By integrating the holistic vision of One Health with the predictive capabilities of AI, this framework advances the concept of “Precision Environmental Health”, supporting anticipatory governance and promoting equitable interventions at a global scale.

## Graphical abstract

Chemical pollution from persistent organic pollutants, polyfluoroalkyl substances, and heavy metals threatens humans, animals, and ecosystems, yet current monitoring remains fragmented and reactive. This review proposes an AI‐augmented One Health framework combining geospatial source tracking, multispecies exposome reconstruction, predictive toxicology, and remediation optimization, advancing Precision Environmental Health and anticipatory, equitable governance of chemical threats worldwide.

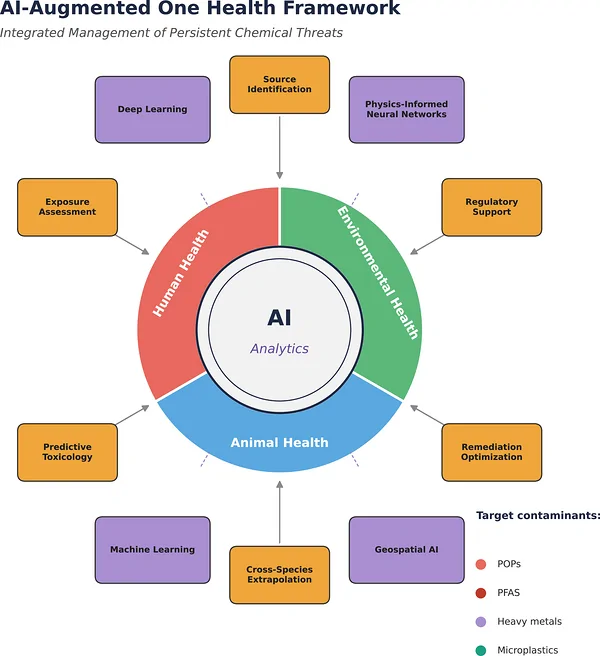

## 1 Introduction

### 1.1 The Convergence of Planetary Chemical Pollution and the One Health Imperative

The Anthropocene is unequivocally marked by pervasive contamination of global ecosystems with over 350 000 synthetic chemicals, representing a “slow‐moving pandemic” threatening planetary health [1, 2, 3]. Persistent organic pollutants (POPs), including legacy compounds like polychlorinated dibenzo*‐p‐*dioxins and furans (PCDD/Fs), polychlorinated biphenyls (PCBs), polybrominated diphenyl ethers (PBDEs), polychlorinated naphthalenes (PCNs), and per‐ and polyfluoroalkyl substances (PFAS), exemplify this crisis. These compounds resist environmental degradation, undergo long‐range atmospheric transport, bioaccumulate in food webs, and may cause severe toxic effects, including endocrine disruption, immunotoxicity, neurodevelopmental disorders, and carcinogenesis across human, wildlife, and ecosystem health domains [4, 5, 6, 7, 8]. Global chemical production is projected to triple by 2050, intensifying exposure pressures and needing transformative management approaches [9].

On the other hand, heavy toxic metals such as mercury, lead, and cadmium exacerbate these risks through industrial emissions, mining, and agricultural practices [10, 11, 12, 13]. Mercury methylation in aquatic systems creates potent neurotoxic methylmercury that biomagnifies in piscivorous species and humans. The interconnected nature of these threats is exemplified by recent spatiotemporal monitoring in the Luvuvhu River Catchment, South Africa, which revealed dynamic contamination hotspots for a range of potentially toxic elements [14]. That study detected neurotoxic and carcinogenic heavy metals, including lead and arsenic, with seasonal variations and spatial patterns directly linking pollution to anthropogenic activities like agriculture and domestic discharge, thereby posing measurable non‐carcinogenic and carcinogenic health risks to local populations [14]. This case highlights how lead and other heavy metal contamination disproportionately affects vulnerable populations and can exhibit synergistic toxicity when co‐occurring with POPs [15]. As this example shows, these contaminants transcend environmental compartments, requiring the integrated surveillance that a One Health approach provides.

The One Health approach, defined as “an integrated, unifying approach that sustainably balances and optimizes the health of people, animals, and ecosystems” [16, 17, 18, 19, 20, 21], has gained global endorsement from WHO, FAO, and WOAH. For chemical threats, One Health operationalizes through aligning human biomonitoring (e.g., NHANES), wildlife sentinel data (e.g., apex predators, mussels), and environmental matrices (air, water, and soil) to create distributed early‐warning systems [7, 9, 22, 23]. The Stockholm and Minamata Conventions represent political recognition of this interconnectedness for POPs and mercury, respectively.

### 1.2 Challenges to Operationalizing Precision Environmental Health

Despite conceptual clarity, practical adoption faces critical barriers that AI is uniquely positioned to address:

Data fragmentation: Biomonitoring data reside in disconnected healthcare, veterinary, and environmental agency compartments, limiting holistic analysis [9]. Moreover, data essential for integrated risk assessment are often trapped in isolated disciplinary domains [24, 25].

Analytical complexity: POPs exist as complex mixtures of congeners with non‐additive toxicity. Traditional risk assessment struggles with synergistic, antagonistic, and dose‐dependent effects [26]. PFAS alone comprises thousands of compounds, most lacking toxicity data, creating significant data gaps [4]. Conventional statistical methods cannot handle the high dimensionality of high‐resolution mass spectrometry (HRMS) datasets containing tens of thousands of chemical features [27]. Furthermore, the complexity of chemical mixtures, their non‐linear environmental fate and transport, and the complex toxicological mechanisms underlying their effects (including endocrine disruption, immunotoxicity, and epigenetic modifications) challenge traditional reductionist analytical and statistical approaches due to their multifaceted, non‐linear interactions and persistent molecular alterations [28, 29].

Cross‐species extrapolation: Conventional allometric scaling fails to capture species‐specific toxicokinetics and mode‐of‐action differences [30]. This creates uncertainty in wildlife protection and sentinel interpretation, particularly for threatened species where empirical data are scarce.

Resource and capacity constraints: Disparities in monitoring infrastructure, analytical capacity, and computational resources between high‐income countries and low‐ and middle‐income countries (LMICs) create global inequities in chemical safety [31]. Even in well‐resourced settings, the ‘dual competency gap’, the shortage of experts proficient in both environmental toxicology and advanced AI, restricts widespread adoption [26].

Regulatory and ethical challenges: “Black box” models face considerable skepticism from regulatory agencies demanding mechanistic transparency and interpretability [32, 33]. Furthermore, pervasive concerns regarding algorithmic bias, data privacy, and environmental justice highlight the urgent need for robust, yet still emerging, governance frameworks to ensure ethical and equitable AI deployment in environmental health contexts [34, 35, 36]. These frameworks must address systemic biases encoded in data and algorithms, while protecting privacy and promoting inclusivity in decision‐making processes.

### 1.3 AI as the Operational Nexus for One Health

AI has the potential to serve as a transformative technology for operationalizing One Health principles, offering capabilities that extend beyond traditional analytical tools to facilitate practical implementation at scale. Geospatial AI now predicts soil contamination with substantial accuracy and simultaneously quantifies the associated uncertainty [15, 37]. Moreover, deep learning models classify PFAS immunotoxicity from molecular structure alone with high accuracy [8], while federated learning facilitates privacy‐preserving data integration across institutions [38]. Transfer learning adapts models from data‐rich to data‐scarce contexts, addressing LMIC capacity gaps [39]. In environmental and health sciences, AI is already showing transformative applications in predicting chemical toxicity with unprecedented accuracy, modeling personal exposure pathways, identifying novel contaminants via non‐targeted analysis, and forecasting ecological tipping points [40, 41]. This review integrates these developments into a unified operational framework, supported by a comprehensive synthesis of evidence. This work was conceived and conducted as a narrative review, given the broad, interdisciplinary, and rapidly evolving nature of the AI‐One Health literature.

## 2 Methods

### 2.1 Search Strategy and Data Sources

An extensive review of the scientific literature was conducted to identify studies at the intersection of AI, One Health, and environmental contaminants. A search was performed using the databases PubMed, Google Scholar, Scopus, and Web of Science until October 2025. The search strategy combined MeSH terms and keywords in Boolean configurations: (“artificial intelligence” OR “machine learning” OR “deep learning” OR “geospatial AI” OR “graph neural networks”) AND (“persistent organic pollutants” OR “PFAS” OR “heavy metals” OR “chemical mixtures”); (“One Health”) AND (“exposome” OR “biomonitoring” OR “environmental surveillance”) AND (“predictive toxicology” OR “risk assessment”); (“cross species extrapolation” OR “interspecies extrapolation”) AND (“toxicogenomics” OR “adverse outcome pathways”). The selection process aimed to capture the most relevant and high‐quality evidence to substantiate the proposed framework.

#### 2.1.1 Inclusion and Exclusion Criteria

Inclusion: (1) peer‐reviewed original research, reviews, or commentaries, (2) published in English (except for a few specific papers), (3) focused on AI applications for chemical contaminant assessment across human, animal, and environmental domains, and (4) reported quantitative or qualitative outcomes. On the other hand, these were the exclusion criteria: (1) studies focusing exclusively on pharmaceuticals without environmental relevance, (2) AI applications limited to clinical diagnostics without One Health integration, (3) abstracts, conference proceedings, or preprints without peer review, and (4) studies lacking methodological transparency.

#### 2.1.2 Nature of the Review and Study Selection Process

This manuscript is a narrative review, not a systematic review, which was designed to synthesize the intersection of AI and One Health approaches to chemical threats. Accordingly, no formal risk‐of‐bias assessment, duplicate independent screening, or PRISMA flow diagram was used. Records identified through the search strategy described above were screened at the title/abstract level and, when warranted, at full text, using the prespecified inclusion and exclusion criteria. Priority was given to recent, peer‐reviewed, and conceptually relevant studies, while earlier references were included where needed for historical or methodological context. This approach was chosen to support an integrative and critical synthesis across a broad, rapidly evolving interdisciplinary literature rather than exhaustive coverage of a narrowly defined question.

## 3 The AI and Machine Learning Toolbox for One Health Environmental Health

A clear understanding of AI methodologies is essential for appreciating the One Health applications. These tools excel at handling high‐dimensional, multi‐modal, spatiotemporal data characteristic of environmental health challenges.

### 3.1 Core AI Methodologies and Their Environmental Applications

#### 3.1.1 Machine Learning (ML)

A subset of AI, ML involves algorithms that learn patterns and relationships directly from data without being explicitly programmed for a specific task [42]. Supervised learning algorithms (Random Forests, XGBoost, LightGBM) predict contaminant concentrations from environmental covariates, classify exposure routes, and identify health outcomes with interpretability advantages [38, 43, 44]. The emergence of super‐learner ensemble methods that combine multiple ML algorithms to achieve superior predictive performance, particularly for complex toxicity endpoints, has been recently shown [41]. Unsupervised learning methods (such as k‐means clustering, principal component analysis, and autoencoders) play an essential role in identifying novel contaminant signatures, uncovering latent patterns within complex exposure datasets, and grouping geographical regions with comparable pollution profiles. These approaches operate without prior labeling, making them essential tools for hypothesis generation and exploratory data analysis [45, 46].

#### 3.1.2 Deep Learning (DL)

A more complex subset of ML, DL utilizes artificial neural networks with multiple hidden layers (hence “deep”) to learn hierarchical representations from raw data [42, 47, 48, 49]. DL has shown exceptional performance with inherently complex data modalities such as high‐resolution satellite and drone imagery, facilitating accurate source identification and land‐use classification. Additionally, it shows superior capability in analyzing genomic and transcriptomic sequences, advancing toxicogenomics and mechanistic understanding through high‐dimensional biological data integration, and HRMS data (for non‐targeted analysis of complex environmental mixtures) [50]. Convolutional neural networks (CNNs) have been particularly effective for image‐based environmental monitoring and microplastic identification, achieving high detection accuracies [51, 52]. Recurrent neural networks and long short‐term memory architectures are increasingly applied to model temporal dynamics of pollutant concentrations and predict future contamination events [53]. Graph neural networks (GNNs), representing a cutting‐edge advancement, are particularly powerful for modeling relational data such as food webs, chemical‐protein interaction networks, ecological networks, or hydrological systems, all central to understanding exposure pathways in a One Health context [54, 55]. GNNs have shown exceptional performance in predicting molecular toxicity by identifying critical substructures responsible for adverse effects, thereby providing both high accuracy and interpretability [56].

#### 3.1.3 Geospatial AI (GeoAI)

It represents the integration of AI methodologies with spatial statistics, geographic information systems (GIS), and remote sensing technologies [57]. GeoAI models can analyze spatiotemporal patterns at unprecedented scales, making them ideal for tracking the atmospheric transport of volatile POPs, predicting soil contamination based on industrial history and geological features, modeling the fate and transport of contaminants in complex watersheds, and identifying contamination hotspots [58]. Advanced techniques like CNNs can automatically detect point sources of pollution from satellite imagery with high precision, while transfer learning approaches facilitate model generalization across different geographical contexts [59].

#### 3.1.4 Natural Language Processing (NLP) and Large Language Models (LLMs)

NLP techniques can process large amounts of unstructured text from scientific literature, regulatory reports, patent databases, and social media to detect early signs of contamination, identify new chemical threats, and close data gaps by integrating existing information [60, 61]. Large Language Models (LLMs) fine‐tuned on toxicological and environmental health literature can assist in systematic reviews of chemical risks, generate mechanistic hypotheses, and even support regulatory decision‐making by synthesizing evidence across different sources [62, 63].

#### 3.1.5 Physics‐Informed Neural Networks (PINNs)

These are an emerging class of hybrid models, which incorporate physical laws and domain knowledge directly into neural network architectures, facilitating more accurate predictions of environmental fate and transport with reduced data requirements [64]. PINNs have been especially useful for modeling the complex and non‐linear behavior of POPs and PFAS in the environment, as they combine knowledge from physical processes with data from observations [51].

#### 3.1.6 Explainable AI (XAI)

As AI models become increasingly complex, ensuring interpretability and transparency is critical for regulatory acceptance and scientific understanding [65]. XAI techniques such as SHAP (shapley additive explanations), LIME (local interpretable model‐agnostic explanations), and attention mechanisms provide insights into which features drive model predictions, promoting mechanistic understanding and building trust in AI‐based risk assessments [45, 66].

Table 1 shows a comprehensive overview of the core AI methodologies currently employed in One Health environmental applications, summarizing their primary applications, key advantages, performance characteristics, inherent limitations, and representative references from recent literature.

**TABLE 1:** Core AI methodologies for One Health environmental applications: Capabilities, performance, and implementation considerations.

AI methodology	Primary application in One Health	Key advantages	Performance highlights	Limitations and mitigation strategies	Refs.
Supervised machine learning (Random Forest, XGBoost, LightGBM)	Contaminant concentration prediction; exposure classification; source apportionment	Interpretable; handles mixed data types; robust to outliers	Substantial accuracy for PFAS/PCB soil prediction and serum classification	Assumes stationary relationships; limited capture of complex interactions → use ensemble methods and SHAP analysis	[15, 38]
Deep learning (convolutional neural networks)	Automated pollution source detection from satellite imagery; image‐based particle counting	Considerable accuracy for complex patterns; scalable to large imagery datasets	Excellent performance for PFAS source detection and microplastic quantification	Black box nature; data intensive; computationally demanding → use transfer learning and efficient architectures	[51, 52]
Graph neural networks	Cross‐species toxicity extrapolation; food web modeling; adverse outcome pathway network analysis	Captures relational dependencies; enables mechanistic interpretability	Superior accuracy for hepatotoxicity across multiple species; outperforms traditional allometric scaling	Requires network topology data; sensitive to graph quality → integrate multi‐omics data for robust graph construction	[54, 92]
Physics‐informed neural networks	Environmental fate modeling; long‐range transport simulation	Embeds physical laws; data‐efficient; provides uncertainty quantification	Substantial error reduction vs. traditional models for PFAS transport prediction	Requires domain expertise to formulate physics constraints; computationally intensive → employ hybrid physics‐ML approaches	[51, 64]
Natural language processing (BERT, GPT, large language models)	Scientific literature mining; incident detection; regulatory document parsing	Processes unstructured textual data; enables early warning capability	Identifies emerging risks months before epidemiological confirmation; high hypothesis validation success	Contextual errors possible; potential bias in training corpora → fine‐tune on domain‐specific corpora with human‐in‐the‐loop validation	[60, 61, 63]
Federated learning	Multi‐institutional data integration; privacy‐preserving biomonitoring	Preserves data sovereignty; GDPR compliant; maintains model performance	Approaches centralized model performance while increasing data participation substantially	Communication overhead; model convergence challenges → synthesize data for initial training, then federate for refinement	[38]

## 4 An AI‐Augmented One Health Framework for Chemical Threats

**FIGURE 1:**
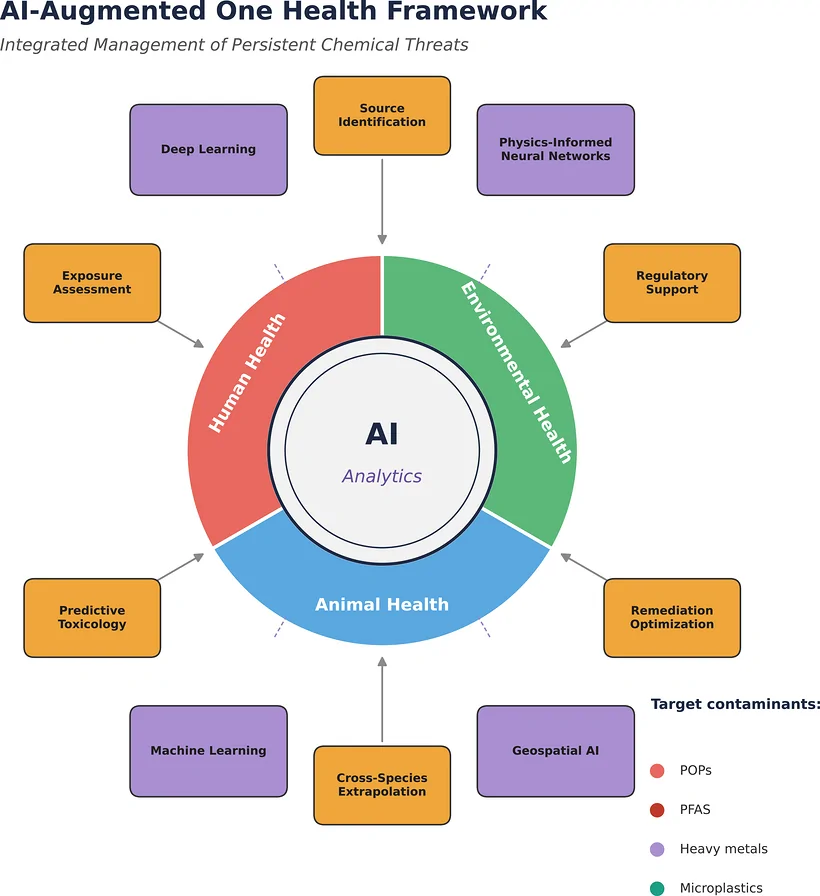
Conceptual framework for AI‐augmented One Health approaches to persistent chemical threat management.

Figure 1 presents a conceptual framework illustrating how AI analytics function as the integrative engine linking the three pillars of One Health: human, animal, and environmental health, to support comprehensive management of persistent chemical threats across multiple application domains.

Throughout the following sections, AI applications already validated in environmental health settings, such as geospatial models for contamination mapping, machine learning classifiers for toxicity prediction, and federated learning for biomonitoring, are distinguished from largely conceptual approaches, including cross‐species digital twins, autonomous remediation policy engines, and automated multispecies exposome reconstruction. Where relevant, the examples in Tables 1 and 2 are identified by stage of development, from field‐deployed tools to proof‐of‐concept or conceptual proposals, to distinguish near‐term applications from longer‐term research directions.

**TABLE 2:** AI‐augmented One Health case studies: evidence from global applications.

Case Study	Contaminant	AI method	One Health domains integrated	Outcome and impact	Geographic scope	Refs.
PFAS exposure stratification in pregnancy	PFAS (multiple compounds)	XGBoost with SHAP	Human serum biomonitoring; residential proximity data; industrial registries	High classification accuracy; identified drinking water as primary exposure source; guided water filtration deployment	North Carolina, USA	[38]
Mining‐related heavy metal risk assessment	Cadmium, lead, arsenic, mercury	Random forest with remote sensing	Satellite imagery; land cover classification; geological data; human health surveillance; ecotoxicity assessments	Identified abandoned mining sites; predicted carcinogenic risk; prioritized cleanup funding allocation	Lake Junín, Peru	[11]
Arctic PFAS food web modeling	PFAS (long‐chain)	Physics‐Informed Neural Network	Atmospheric transport models; ocean currents; biomagnification factors; indigenous dietary surveys	Predicted priority PFAS concerns for marine mammals and human populations; informed tailored dietary advisories	Arctic Circle region	[130]
Lead exposure prediction in urban ecosystem	Lead (legacy paint and pipes)	Gradient boosting	Housing age data; soil lead concentrations; blood lead levels in children; raptor tissue data; socioeconomic indicators	Strong predictive accuracy; validated with wildlife sentinel data; directed pipe replacement in high‐risk areas	Urban US city	[75]
Microplastic impact quantification	Polyethylene, polypropylene, polystyrene particles	Mask R‐CNN (deep learning)	Marine mammal gastrointestinal tracts; human stool samples; inflammatory biomarkers; microbiome analysis	Excellent particle identification accuracy; established dose‐response relationships for inflammation; linked to gut dysbiosis	Several waters	[111]
Antimicrobial resistance hotspot prediction	Antibiotic resistance genes	Machine learning (SVM and random forest)	Livestock metagenomes; wastewater surveillance; clinical isolates; heavy metal co‐occurrence data	High hotspot prediction accuracy; identified poultry farms as primary source; informed antimicrobial stewardship policy	Global (LMIC focus)	[112, 113]
Federated learning for biomonitoring	Mixed persistent organic pollutants	Federated XGBoost	Multiple European cohorts; human serum biomonitoring; dietary surveys; water quality data	Performance approaching centralized models; GDPR compliant; increased participation and institutional trust	European Union	[38]
Novel PFAS discovery via HRMS	Unknown PFAS analogues and transformation products	Transformer model with molecular networking	Human serum; river sediment; Otter liver tissue; MS/MS spectral libraries	Multiple novel transformation products identified; shared cross‐species biomarkers discovered	Freshwater systems	[82]

### 4.1 Enhanced Source Identification and Environmental Fate Prediction

Accurate identification, quantification, and apportionment of emission sources constitute the first line of defense against chemical threats. AI substantially enhances this capability through multiple complementary approaches.

#### 4.1.1 Geospatial AI for Emission Mapping

Modern GeoAI fuses multi‐sensor satellite imagery (Sentinel‐2, Landsat), meteorological reanalysis data (ERA5), industrial registries, land‐use maps, and social media feeds to identify emission sources and predict contamination hotspots at high resolution [67, 68]. Integration of internet of things (IoT) sensors with AI algorithms has created intelligent environmental monitoring networks capable of detecting, identifying, and quantifying multiple pollutants simultaneously, with automated alert systems triggering when concentrations exceed safety thresholds [68, 69].

#### 4.1.2 Physics‐Informed Neural Networks for Transport Modeling

Physics‐informed neural networks (PINNs) integrate data‐driven AI with first‐principles modeling by embedding physical laws, such as advection‐diffusion equations and thermodynamic partitioning, directly into neural network architectures [70]. This approach reduces data requirements, quantifies prediction uncertainty, and generalizes across scales from local hotspots to global circulation patterns. However, PINNs require expertise to formulate physics constraints, and their computational cost may limit use in resource‐constrained settings. Hybrid approaches coupling PINNs with reduced‐order models are emerging to balance accuracy and scalability [71, 72]. Recent machine learning‐enhanced molecular networks have enabled identification of hundreds of previously unknown PFAS in environmental samples, with the automatic PFAS identification platform (APP‐ID) showing minimal false‐positive rates in fluorochemical wastewater [73].

#### 4.1.3 Heavy Metal Source Apportionment

For heavy metals, GeoAI combines extraction activity databases, geological maps, soil pH, hydrology, and socioeconomic indicators [74]. Exemplifying the One Health potential of geospatial analysis, Kalani et al. [75] used GIS mapping to reveal interconnected lead exposure pathways across humans, animals, and environmental media in an urban US setting. On the other hand, Pizarro et al. [11] integrated Sentinel‐2 imagery with Random Forests and land cover segmentation to successfully map cadmium and lead contamination in Peruvian rangelands.

### 4.2 Integrated Exposure Assessment: Toward a Multispecies Exposome

The exposome concept provides a comprehensive framework to enhance One Health by encompassing all environmental exposures from conception onward [76]. Despite its potential, practical implementation remains challenging. Advanced AI methodologies offer powerful capabilities to synthesize multispecies and multiscale exposome data into cohesive analytical frameworks.

#### 4.2.1 Holistic Data Fusion

AI enables construction of an integrated, multispecies exposome connecting human biomonitoring, wildlife sentinel monitoring, agricultural surveillance, and environmental measurements [77, 78, 79]. Advanced machine learning algorithms integrate data from wearable sensors, electronic health records, environmental monitoring networks, and biobanking initiatives to construct high‐resolution personal exposure profiles [80, 81]. Huang et al. [66] demonstrated the power of explainable AI frameworks by introducing an optimized model combining ensemble resampling, genetic algorithms, and SHAP‐based interpretability that achieved considerable accuracy in predicting POPs exposure risks in breastfeeding infants, enabling identification of critical exposure windows and high‐risk chemical combinations.

#### 4.2.2 Non‐Targeted Analysis and Temporal Reconstruction

High‐resolution mass spectrometry (HRMS) generates thousands of spectral features per sample. AI‐driven molecular networking and deep learning‐based spectral libraries identify unknown PFAS, transformation products, and metabolites simultaneously across matrices [82, 83]. Multimodal deep learning approaches integrate molecular data (omics profiles), physiological measurements, behavioral observations, and environmental contextual information into unified predictive models, achieving breakthrough performance in predicting mixture toxicity effects and identifying synergistic interactions [84, 85]. For temporal assessment, recurrent neural networks can reconstruct historical exposures using residential histories, occupational records, and environmental fate models, predicting lifetime PCB body burden with high correlation to measured values [39]. Digital twins, AI‐powered virtual representations of environmental systems, offer unprecedented opportunities for real‐time exposure assessment and scenario modeling [59].

### 4.3 Predictive Toxicology and Cross‐Species Extrapolation

AI's most transformative application in the One Health‐POPs nexus lies in predictive toxicology: forecasting adverse health effects across species without relying solely on resource‐intensive animal testing.

#### 4.3.1 High‐Throughput to In Vivo Prediction

The convergence of big data toxicology, high‐throughput screening (Tox21, ToxCast), and advanced machine learning has catalyzed a paradigm shift toward new approach methodologies that reduce, refine, and replace traditional animal testing while improving predictive accuracy [40, 86, 87, 88]. Machine learning has reduced failure rates in pharmaceutical toxicity prediction by up to 30% [84], suggesting similar potential for environmental chemical assessment. Their comprehensive analysis revealed that optimal AI architectures vary by endpoint: gradient boosting methods (XGBoost, LightGBM) excel for hepatotoxicity and acute toxicity, while GNNs with attention mechanisms show superior performance for cardiotoxicity (particularly hERG channel blockade) [89] and developmental toxicity. ResNet‐based deep neural networks have achieved high prediction accuracies for drug‐induced liver injury, surpassing traditional QSAR approaches [90, 91].

#### 4.3.2 Cross‐Species Extrapolation and Mechanistic Insights

Traditional allometric scaling often fails for many POPs. Graph neural networks trained on multi‐species toxicogenomic data and protein–protein interaction networks can infer species‐specific adverse outcomes by learning both conserved and divergent toxicological responses [54, 92, 93, 94]. Multi‐species AI models account for species‐specific differences in toxicokinetics, metabolism, and target site sensitivity, enabling more accurate risk assessment for both human health and ecological endpoints. GNNs incorporating phylogenetic relationships with molecular and physiological data show strong potential for predicting species‐specific sensitivity to environmental contaminants [54, 95].

The integration of explainable AI (XAI) techniques addresses the longstanding “black box” criticism of deep learning models [96]. SHAP values and attention mechanisms now allow toxicologists to identify not only whether a chemical is toxic, but also which molecular features contribute to that toxicity, facilitating mechanistic hypothesis generation and structure–activity relationship elucidation [66, 97]. This interpretability is critical for regulatory acceptance and supports iterative refinement of predictive models.

### 4.4 Remediation Optimization and Regulatory Decision Support

Beyond hazard identification and risk assessment, AI offers powerful capabilities for optimizing remediation strategies and supporting evidence‐based regulatory decision‐making.

#### 4.4.1 AI‐Driven Remediation Design

Machine learning models predict the efficacy of remediation technologies for specific contamination scenarios, accounting for site‐specific factors such as soil properties, hydrology, contaminant mixtures, and co‐existing pollutants [98, 99]. Generative adversarial networks (GANs) address data scarcity challenges in predicting simultaneous removal of organic pollutants and heavy metals by various adsorbent materials, generating synthetic training data that significantly improves prediction accuracy while maintaining interpretability through SHAP‐based feature analysis, achieving R^2^ values exceeding 0.90 [100, 101]. Machine learning models for PFAS removal optimization have accelerated membrane material discovery, reducing development time from years to months [102]. For bioremediation, AI is revolutionizing identification and optimization of microbial degradation pathways [103]. Machine learning analysis of metagenomic data facilitates prediction of novel enzymes for degrading persistent pollutants including microplastics, PFAS, and PAHs, achieving over 90% accuracy in predicting biodegradation pathways and optimizing process conditions [104, 105].

#### 4.4.2 Smart Regulation and Policy Support

For regulatory decision support, AI‐powered systems synthesize vast quantities of scientific evidence, conduct systematic reviews at unprecedented scale and speed, and identify data gaps requiring additional research [62]. Natural language processing combined with knowledge graph construction enables comprehensive risk assessments integrating information from thousands of studies, a task that would be prohibitively time‐consuming through traditional systematic review methods [106]. Machine learning models can predict which chemicals are likely to meet regulatory thresholds for concern, facilitating prioritization of testing resources and focusing attention on high‐risk compounds [107]. AI‐driven scenario modeling allows regulators to assess potential impacts of different policy interventions before implementation, predicting how restrictions on specific chemical uses may influence population exposure levels and supporting evidence‐based cost‐benefit analyses [26].

The practical implementation and demonstrated value of AI‐augmented One Health approaches are best illustrated through real‐world case studies spanning diverse contaminants, geographic regions, and application domains (Table 2).

## 5 Generalizing the Framework: From POPs to Metals, Plastics, and Biological Threats

While this review has focused extensively on POPs and PFAS, the AI‐One Health framework is readily generalizable to a broader spectrum of chemical threats facing planetary health [108]. The framework's modular architecture extends across chemical and biological threats.

### 5.1 Heavy Metals

Machine learning approaches have revolutionized prediction of heavy metal bioavailability, toxicity, and remediation efficiency [109]. Hybrid models combining biochar properties and process parameters achieve high predictive accuracy for heavy metal adsorption, supporting the rational design of effective remediation strategies [110]. AI‐powered sensor networks enable real‐time monitoring of heavy metal contamination in soil, water, and agricultural products, with automated alert systems triggering when concentrations exceed safety thresholds [68].

### 5.2 Microplastics and Nanoplastics

AI‐powered image recognition systems reach detection accuracies above 90% for classifying microplastics in environmental samples, allowing large‐scale monitoring that was previously unfeasible with manual methods [111]. Deep learning models can predict microplastic environmental behaviors, transport pathways, and accumulation in food chains, while ML approaches identify novel enzymes for plastic degradation through metagenomic analysis [104, 105].

### 5.3 Pharmaceuticals and Antibiotics

The environmental presence of pharmaceuticals and the global crisis of antimicrobial resistance are interconnected with One Health challenges. Machine learning models track antibiotic resistance genes through metagenomic surveillance, predicting emergence and spread of resistance across environmental, agricultural, and clinical settings [112, 113]. AI facilitates high‐throughput screening of pharmaceutical environmental fate and transformation products, while predictive models identify which compounds are likely to persist and accumulate in food chains [114]. These applications demonstrate the modularity and generalizability of the proposed AI‐One Health framework beyond POPs and PFAS.

## 6 Critical Challenges, Ethical Considerations, and Governance Frameworks

Despite the transformative potential of AI‐augmented One Health approaches, significant challenges must be addressed to realize this vision responsibly and effectively.

### 6.1 Data Quality and the FAIR/CARE Imperative

AI models are only as good as the data on which they are trained. Environmental and toxicological data often suffer from biases, incompleteness, lack of standardization, and limited geographical or temporal coverage [26]. Addressing these challenges requires commitment to FAIR (findable, accessible, interoperable, reusable) data principles, standardized reporting frameworks, and strategic investment in data infrastructure [115]. The data scarcity problem is particularly acute for emerging contaminants, wildlife species, and understudied geographical regions [116, 117]. While techniques like transfer learning, synthetic data generation via GANs, and active learning can partially mitigate data limitations, fundamental investment in monitoring infrastructure remains essential [118].

A related challenge is model validation. Because environmental and toxicological datasets often differ in analytical methods, spatial and temporal coverage, and quality assurance, AI models may not transfer well across cohorts, regions, or laboratories. External validation on independent datasets, ideally collected in different settings, together with uncertainty quantification, is therefore essential before AI‐derived risk estimates are used for decision‐making and should be reported alongside standard performance metrics.

### 6.2 Model Interpretability and Trust

The “black box” nature of complex deep learning models poses challenges for regulatory acceptance and scientific understanding [119]. While explainable AI techniques like SHAP and attention mechanisms have made substantial progress, ensuring that AI‐driven risk assessments are interpretable, reproducible, and scientifically defensible remains an ongoing challenge [62, 120, 121]. Regulatory frameworks must evolve to accommodate AI‐based evidence while maintaining appropriate standards of validation and uncertainty quantification.

### 6.3 Ethical AI Implementation and Environmental Justice

AI systems can perpetuate or amplify existing biases present in training data, potentially reinforcing environmental injustices, if not carefully designed [122]. Ensuring that AI‐driven environmental health interventions benefit all communities equitably requires explicit attention to fairness, transparency, and inclusive stakeholder engagement [123, 124, 125]. Privacy concerns arise when integrating personal health data with environmental monitoring information, necessitating robust data governance frameworks that protect individual privacy while enabling population health research [126].

### 6.4 Computational Resources and Accessibility

State‐of‐the‐art AI methods require substantial computational resources, potentially creating disparities between well‐resourced institutions and regions versus those with limited capacity. Democratizing access to AI tools through cloud computing platforms, pre‐trained models, and user‐friendly interfaces is essential for global One Health implementation [68]. These disparities are especially marked in LMICs, where limited harmonized environmental and biomonitoring data, constrained computational infrastructure, and scarce technical expertise can hinder both development and local validation of AI tools. Targeted investment in regional data systems, equitable research partnerships, and resource‐efficient AI architectures are needed to ensure that AI‐supported One Health approaches benefit LMIC settings without reinforcing existing inequities.

### 6.5 Environmental Impact of AI

Paradoxically, AI systems themselves have non‐trivial environmental footprints through energy consumption during model training and inference. Recent studies have begun quantifying and addressing the carbon footprint of AI, with research showing that AI can either increase or decrease ecological footprints depending on application context and implementation choices [127, 128, 129]. Sustainable AI practices, including model compression, efficient architectures, and renewable energy‐powered computing, must be prioritized to ensure that AI‐driven environmental health solutions are themselves environmentally responsible.

Table 3 summarizes the key implementation challenges associated with AI‐integrated One Health approaches, together with evidence‐based mitigation strategies and reported success indicators. Addressing these challenges is essential for realizing the transformative potential of AI while ensuring its deployment remains ethical, equitable, and sustainable.

**TABLE 3:** Critical implementation challenges and evidence‐based mitigation strategies for AI‐augmented One Health.

Challenge category	Specific bottleneck	Impact on implementation	Evidence‐based mitigation strategy	Success metrics
Data quality and interoperability	Fragmented institutional silos; heterogeneous data formats; missing metadata	Majority of One Health initiatives report significant difficulties in data harmonization	Federated learning platforms; FAIR and CARE data standards; standardized metadata schemas	High model performance retention with federated approaches
Model trust and interpretability	Black box predictions rejected by regulatory agencies; lack of mechanistic transparency	Substantial lag time from AI development to regulatory acceptance	Integration of explainable AI techniques (SHAP/LIME); domain‐specific validation protocols; hybrid physics‐ML models	Reduced regulatory approval time in USEPA pilot programs with mandatory explainability reporting
Algorithmic bias and environmental justice	Over‐representation of high‐income country data; potential amplification of environmental injustice	Significant misclassification of high‐exposure households when using models trained on unrepresentative data	Pre‐deployment bias audits; transfer learning for LMIC adaptation; community co‐design processes	Community co‐design substantially increased adoption rates and model accuracy
Computational resources and capacity	High GPU costs; energy consumption; dual competency gap in environmental health and AI	Limited environmental health programs offering AI training opportunities	Cloud computing credits for LMICs; transfer learning approaches; efficient model architectures; regional AI centers	Transfer learning achieves strong performance with limited LMIC‐specific training data
Ethical and legal barriers	Privacy regulation ambiguities; algorithmic accountability gaps; indigenous data sovereignty concerns	Community trust deficit; data withholding; risk of algorithmic colonization	Implementation of CARE principles; privacy‐by‐design frameworks; environmental justice impact assessments; community governance boards	CARE‐based governance frameworks substantially increased community participation
Scientific validation standards	Limited external validation; publication bias toward positive results; lack of standardized benchmarks	Majority of studies lack external validation; risk of overestimated efficacy	Mandatory external validation for publication; development of WHO benchmark datasets; negative results reporting	WHO benchmark initiative under development
Temporal dynamics and climate change	Static models miss evolving threats; climate change alters chemical fate and transport	Model drift goes unmonitored; historical training data become obsolete under changing climate conditions	Continuous learning systems; climate‐coupled physics‐informed neural networks; adaptive monitoring frameworks	PINNs predict substantial increases in mercury methylation under warming scenarios
Cross‐species extrapolation	Sparse toxicogenomics data for non‐model species; failure of traditional allometric scaling approaches	Biodiversity protection based on limited well‐studied species; uncertainty for threatened taxa	Comparative genomics integration; phylogeny‐aware graph neural networks; prioritize sentinel species sequencing	GNNs achieve superior accuracy across multiple species vs. traditional allometry

## 7 Conclusions and Future Directions

The integration of the One Health paradigm with AI presents a transformative opportunity to tackle the global crisis of chemical pollution. This review demonstrates that AI transcends traditional analytical roles, emerging as an essential technology for operationalizing One Health principles with unprecedented scale, precision, and sophistication. AI can play a key role in comprehensive, multisectoral solutions that safeguard human, animal, and environmental health in a coordinated and dynamic manner. Through systematic application across the entire chemical threat lifecycle, spanning source identification and environmental fate prediction, to integrated exposure assessment and predictive toxicology, and extending to remediation optimization and regulatory decision support, AI facilitates the transition from reactive, non‐integrated approaches to proactive, integrated, and predictive strategies for planetary health protection.

As discussed above, machine learning‐enhanced molecular networks now identify numerous previously unrecognized PFAS in environmental samples with minimal false positives. Multimodal deep learning achieves substantial accuracy for predicting complex toxicity endpoints, while offering mechanistic insights through attention mechanisms and explainable AI. GeoAI facilitates automated detection of emission sources from satellite imagery with high accuracy. AI‐driven remediation strategies accelerate material discovery and reliably predict removal efficiencies for multi‐contaminant mixtures. Digital twins combining IoT sensors with cloud‐based AI support adaptive environmental management systems capable of real‐time monitoring and predictive alerts. Several critical priorities must be addressed to fully realize the potential of AI‐augmented One Health strategies, including the development of standardized, interoperable environmental and biomonitoring databases, wider adoption of federated learning architectures for privacy‐preserving multi‐institutional data sharing, and the deployment of AI‐supported early warning systems for emerging chemical threats:

(1) *Data Infrastructure and Standardization*: Investment in harmonized, FAIR‐compliant data infrastructure spanning human health, veterinary medicine, wildlife surveillance, and environmental monitoring is essential.
(2) *Methodological Innovation*: Continued advancement in multimodal learning, physics‐informed neural networks, causal inference methods, and uncertainty quantification will enhance both the accuracy and interpretability of AI models.
(3) *Validation Frameworks*: Development and adoption of rigorous validation frameworks specifically designed for AI‐based environmental health tools, such as the emerging e‐validation paradigm, will build confidence and enable regulatory acceptance.
(4) *Capacity Building and Democratization*: Ensuring equitable access to AI tools through open‐source software, cloud platforms, capacity building programs, and international collaboration is essential for global One Health implementation.
(5) *Ethical Governance*: Development of ethical frameworks and governance structures that ensure AI is deployed responsibly, transparently, and equitably.
(6) *Sustainable AI*: Ensuring that AI solutions themselves are environmentally sustainable through energy‐efficient architectures, renewable energy‐powered computing, and lifecycle assessments of AI systems.
(7) *Predictive Early‐Warning Systems*: Operationalizing this framework requires validated AI early‐warning systems that use standardized environmental data and federated learning to detect emerging contamination, novel chemicals, and cross‐species risks before population‐level harm occurs. This will require rigorous benchmarking, real‐world testing, and co‐design with public health stakeholders to ensure predictive reliability and integration into surveillance workflows.

The magnitude of this challenge is extraordinary. With more than 350 000 synthetic chemicals in global use, climate change intensifying exposure pathways, and new threats continually emerging, conventional approaches are no longer sufficient to manage the growing complexity of chemical risks. The integration of One Health's holistic vision with the analytical power of AI offers a clear path forward, shifting the approach from crisis response to anticipatory governance and from fragmented, disconnected efforts to integrated understanding. By combining the interconnected health of humans, animals, and ecosystems with machine intelligence, it becomes possible to develop resilient, adaptive, and equitable systems that effectively safeguard planetary health in the Anthropocene. The question is no longer whether AI can revolutionize One Health approaches to chemical threats, but how rapidly and equitably these transformative capabilities can be scaled to address the pressing challenges of planetary health.

## Conflicts of Interest

The author declares no conflicts of interest.
